# A decision-support tool for funding health innovations at a tertiary academic medical center

**DOI:** 10.1017/S0266462323000028

**Published:** 2023-02-13

**Authors:** Yiying Cai, Nuraini Nazeha, Shamira Perera, Alexandre H. Thiery, Michaël J.A. Girard, Chen E. Lee, Weiwei Hong, Nicholas Graves

**Affiliations:** 1 Programme in Health Services and Systems Research, Duke-NUS Medical School, Singapore, Singapore; 2 Singapore National Eye Centre, Singapore, Singapore; 3 Singapore Eye Research Institute (SERI), Singapore, Singapore; 4 SingHealth Duke-NUS Ophthalmology and Visual Sciences Academic Clinical Programme, Singapore, Singapore; 5 Department of Statistics and Applied Probability, National University of Singapore, Singapore, Singapore; 6 Office for Service Transformation, SingHealth, Singapore, Singapore

**Keywords:** healthcare systems, healthcare evaluation, health technology assessment

## Abstract

**Objectives:**

To report the processes used to design and implement an assessment tool to inform funding decisions for competing health innovations in a tertiary hospital.

**Methods:**

We designed an assessment tool for health innovation proposals with three components: “value to the institution,” “novelty,” and “potential for adoption and scaling.” The “value to the institution” component consisted of twelve weighted value attributes identified from the host institution’s annual report; weights were allocated based on a survey of the hospital’s leaders. The second and third components consisted of open-ended questions on “novelty” and “barriers to implementation” to support further dialogue. Purposive literature review was performed independently by two researchers for each assessment. The assessment tool was piloted during an institutional health innovation funding cycle.

**Results:**

We used 17 days to evaluate ten proposals. The completed assessments were shared with an independent group of panellists, who selected five projects for funding. Proposals with the lowest scores for “value to the institution” had less perceived impact on the patient-related value attributes of “access,” “patient centeredness,” “health outcomes,” “prevention,” and “safety.” Similar innovations were reported in literature in seven proposals; potential barriers to implementation were identified in six proposals. We included a worked example to illustrate the assessment process.

**Conclusions:**

We developed an assessment tool that is aligned with local institutional priorities. Our tool can augment the decision-making process when funding health innovation projects. The tool can be adapted by others facing similar challenges of trying to choose the best health innovations to fund.

## Introduction

Health innovation is any novel idea, product, service, or care pathway with benefits on treatment, diagnosis, education, prevention, or research in healthcare (1). Singapore has a strong track record in health innovation. In the 2020 World Index of Healthcare Innovation, Singapore ranked seventh due to the strong research universities and advances in medical technologies (2). The innovation culture in health care is also evident from the multiple health innovation institutes established locally. This includes the Centre for Healthcare Innovation and the Academic Medicine Innovation Institute.

With the increased number of health innovations, tensions have emerged between nurturing an environment that stimulates innovations and ensuring that scarce resources are used for the most beneficial technologies. Budgets are unable to fund all innovations and choices must be made (3). Hence, a challenge that is increasingly faced by health institutions is how to prioritize health innovations for funding. In Singapore, funding decisions are conventionally decided by a panel of multidisciplinary stakeholders based on their perceived merits of the proposed innovation. However, the perceived merits of an innovation may differ depending on each stakeholder’s motivation (4). For example, physicians often endeavor to achieve optimal care for individual patients. This can be at odds with the “maximal returns on investment” approach of some hospital administrators. In addition, while it is ideally expected that a funded innovation aligns with local and institutional priorities, the process of how this is gauged is rarely made explicit (5).

The Academic Medicine Innovation Institute is a virtual framework jointly managed by SingHealth and Duke-NUS Medical School. The institute aims to convene health innovators across the two institutions and support health innovation efforts in various aspects. In 2021, the institute sought a decision-support tool for prioritizing new competing health innovations for funding. The tool should facilitate the comparison of potentially different health innovations and identify those that support the institute’s priorities in an objective and transparent manner. In this paper, we share our process of developing and piloting of a funding assessment tool to triage new innovation proposals. We demonstrate the use of the assessment tool in funding cycle and provide a worked example of a funded project.

## Methods

### Study setting

SingHealth is Singapore’s largest healthcare cluster and forms a partnership with Duke-NUS Medical School, via an Academic Medicine Centre. The center harnesses each institution’s collective strengths to promote clinical care, education, research, and innovation in health care. In 2020, the center launched the Academic Medicine Innovation Institute to support healthcare innovators. This led to the formation of an Impact Assessment Unit (IAU), which comprises researchers and data analysts who generate and disseminate evidence about the potential value of health innovations. The innovation assessment tool described in this paper was developed as one of the first initiatives of the IAU.

### Description of the innovation assessment tool

We developed the innovation assessment tool using Qualtrics (Provo, UT; see Table 1). Three key components were identified for the assessment tool: “value to the institution,” “novelty,” and “potential barriers to implementation.” These components were suggested after discussion with twelve opinion leaders from the Academic Medicine Centre responsible for synthesizing and setting directions for the innovation landscape within the center.

**Table 1.** Innovation assessment questionnaire.

I. Potential value to the institution							
Question/Statement	Weight	Unweighted score (select one)				Summary of evidence (with references) and justification	Weighted score (Max = 20)
		−1	0	1	2		
*Access.* The innovation directly improves access to services for patients	0.88		Negligible or none at all	Somewhat likely	Strongly		
*Affordability.* The innovation is likely to be ________ to the patient or the healthcare system	0.82		Cost incurring	Cost neutral	Cost saving		
*Capability building.* The innovation has the capability to … (select all that is relevant)^a^	0.82		Build research and/or innovation partnerships	Be scaled beyond the SingHealth Duke-NUS AMC	Be patented or have potential for commercialization		
*Economic productivity.* The innovation is likely to improve the work productivity of patients and or caregivers (e.g., shorter down-time, reduced waiting time).	0.82		Negligible or none at all	Somewhat likely	Strongly		
*Evidence-based.* Is there evidence that the innovation can address the proposed problem?	0.85	Evidence shows detrimental/ negative effect	No evidence	Weak/low level evidence (case reports, retrospective studies)	Strong evidence (systematic review or randomized trial)		
*Health outcomes.* The innovation can directly reduce patient’s morbidity and/or mortality.	0.93		Potential for detrimental impact on morbidity and/or mortality	Modest/ inconclusive impact on morbidity and/or mortality	Likely to reduce morbidity and/or mortality		
*Patient centredness.* The innovation addresses the specific need of the patients and/or caregivers.	0.93		Negligible or none at all	Somewhat likely	Strongly		
*Population impact.* The size of the population that the innovation is likely to impact is …	0.82		Small (<1,000 people)	Medium (1,000 – >100,000 people)	Large (>100,000 people)		
*Prevention.* The innovation is designed to and can prevent future health deterioration or disease occurrence.	0.80		Negligible or none at all	Somewhat likely	Strongly		
*Professional development.* Implementation of the innovation can enable staff to gain new knowledge/skills.	0.58		Negligible or none at all	Somewhat likely	Strongly		
*Safety.* The innovation can improve safety for patients and/or staff.	0.86		Negligible or none at all	Somewhat likely	Strongly		
*Sustainability.* The innovation is likely to be adopted in the healthcare system in an ongoing manner.	0.88		Negligible or none at all	Somewhat likely	Strongly		

II. Novelty					
Question/statement	Answer (choose all applicable)				
Is there evidence that a similar innovation has been developed or deployed within …	None at all	Internationally	Within Asia	In Singapore	In the SingHealth Duke-NUS AMC
Provide information on similar innovation(s) with references.					

III. Potential barriers to implementation			
Question/statement		Answer (select one)	Justification (with references)
Opportunity	*Environmental context and resources.* Are there potential significant limitations in the present resources that may prevent implementation of the innovation (e.g., lack of infrastructural support that cannot be easily rectified)?	Yes/No	
	*Social Influences.* Are there any foreseeable social barriers that may prevent implementation of the innovation?	Yes/No	
Motivation	*Intention/reinforcement.* Does the project team demonstrate a certainty of commitment to bring about the proposed change?	Yes/No	
	*Professional role and identity.* Are the investigators the appropriate group of professionals to bring about the implementation of the innovation?	Yes/No	
	*Beliefs about consequences/optimism.* Are the expectations associated with the innovation reasonable?	Yes/No	
	*Beliefs about capabilities.* Are the healthcare professionals empowered to bring about the implementation of the innovation?	Yes/No	
	*Goals.* Are there proper priority setting and action planning for implementation of the innovation?	Yes/No	
	*Emotions.* Are there potential emotional barriers that might prevent the implementation of the innovation?	Yes/No	
Capabilities	*Knowledge.* Do the innovation require significant advancement of knowledge for the users (e.g. steep learning curve)?	Yes/No	
	*Physical skills.* Do the innovation require specialized skills that might prevent implementation to target users?	Yes/No	

a Each component is allocated a weighted score of 0.82. One or more component can be selected.

Abbreviation: AMC, Academic Medicine Centre.

“Value to the institution” describes how well the innovation will align with the goals of the institution if successfully implemented. “Novelty” described whether the innovation offered a novel solution to the healthcare problem or if substitutes were available. “Potential barriers to implementation” described any significant barriers to implementation that the proposed innovation might face, based on the barriers listed in the Theoretical Domains Framework by Atkins et al. (6). To identify “the value of the institution” attributes, the IAU identified attributes from SingHealth’s recent annual reports. The opinion leaders were then asked to express their relative preference on a Likert scale (from zero = low importance to five = very important) for each attribute so that a weighted scoring system based on mean preferences could be built (see Supplementary Table 1). The maximum possible score for “value to the institution” is twenty. No scores were allocated for the “novelty” or “potential barriers to implementation” component. The developed assessment tool was discussed with the steering committee of the Academic Medicine Innovation Institute and refined according to the comments received.

### Implementation of the innovation assessment tool

We piloted the innovation assessment tool in the Clinical and Systems Innovation Support Main Grant round in May 2021. The grant is a twice-yearly institutional innovation grant with a funding quantum of SGD$100,000 (USD$75,000), aimed at promoting health innovations that can transform health care and support the center’s priorities. To minimize any potential conflict of interest, all IAU researchers were forbidden from participating as a study team member or collaborator from any of the grants that were submitted for assessment.

To complete each assessment, a researcher in the IAU reviewed data from published literature and unpublished preliminary data provided by the innovation teams based on predefined criteria (see Supplementary Table). For the value attributes of “sustainability” and for all attributes in “potential barriers to implementation” component, stakeholders’ input were also sought (see Supplementary Table). Purposive literature search was performed using PubMed, Cochrane Library, Google Scholar, and Google search engine (7). Upon completion, a second IAU researcher would conduct a separate review of the innovation using the same methodology. Any discrepancy in assessment between the two researchers was resolved by discussion. When all assessments were completed, the IAU prepared written reports for each innovation proposal for a final independent panel of assessors (see Supplementary Figure 1). Written feedback was also provided to the individual innovation teams. Using information from the IAU reports and from a 30-minute oral presentation by the innovation teams, the panel of assessors conducted final deliberation and ranked the projects for funding.

## Results

### Innovation assessment timeline

**Figure 1.**
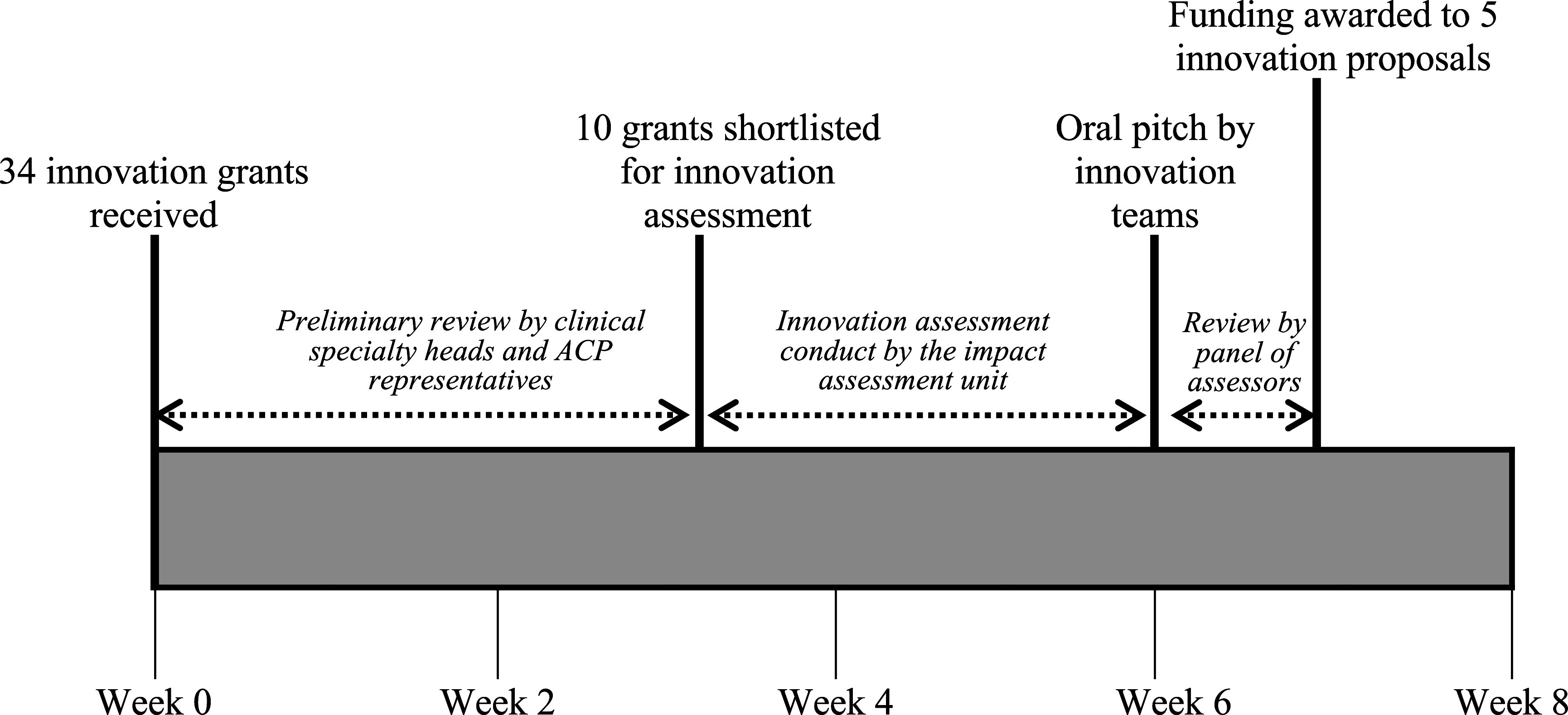
Timeline for review and assessment of innovation proposal. The impact assessment unit used a total of 17 days to review and prepare the written reports of ten innovation proposals.

The timeline for innovation assessment is shown in Figure 1. A total of thirty-four innovation proposals were submitted for potential funding. Ten proposals were supported by clinical specialty heads and the center representatives were shortlisted for innovation assessment. These ten proposals included health innovations comprising multiple aspects including diagnosis, treatment, prevention, service delivery, and education. The IAU used seventeen working days to conduct assessment and prepare the assessment reports for ten proposals. After the assessment, all ten project teams presented their proposed innovations in an oral presentation to a final panel of assessors. Five were selected for funding, based on information from the oral presentations and the assessment reports.

### Results of the innovation assessment

**Figure 2.**
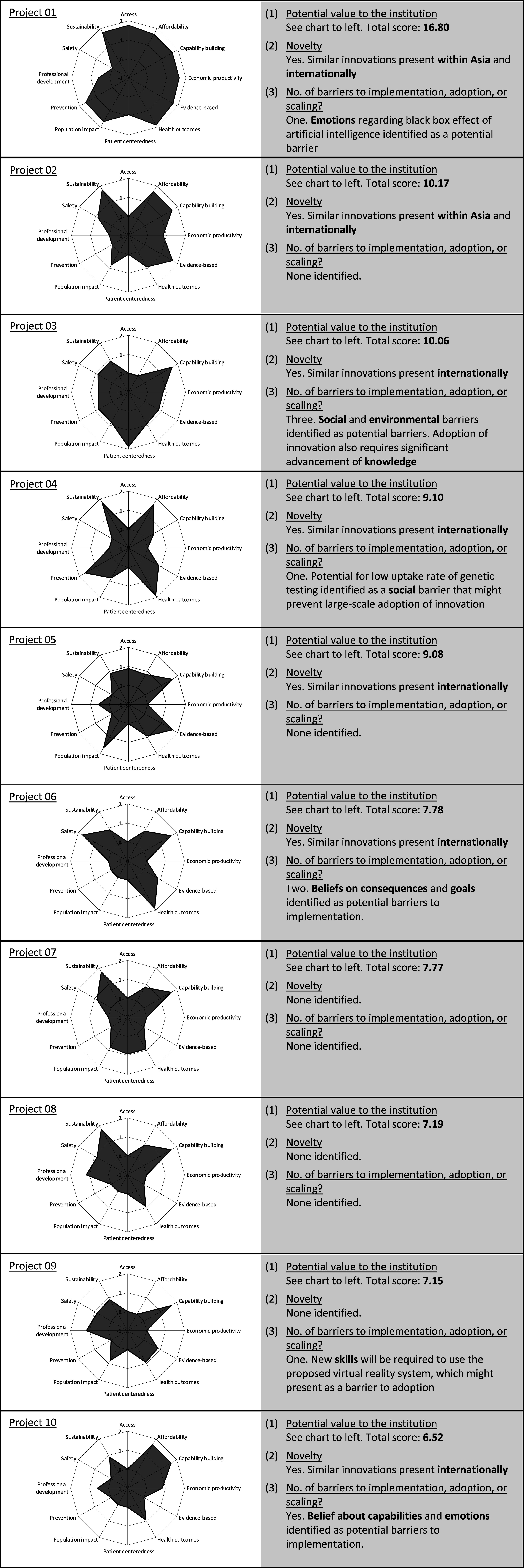
Summary of assessment for ten innovation projects. The different attributes for “value to the institutions” are described by the radar chart (left).

A summary of the innovation assessment conducted by the IAU is presented in Figure 2. Projects that ranked lowest on “value to the institution” were those that scored lower on patient care attributes such as “access,” “patient centeredness,” “health outcomes,” “prevention,” and “safety.” While none of the proposed innovations had potential substitutes within the institution or in Singapore, seven had potential substitutes available regionally or globally. Most of the project teams were able to explain why the substitutes could not be adopted for local use in the oral pitch. At least one potential barrier to implementation was identified in six proposed innovations. Innovation proposal P03 had the highest number of potential barriers (i.e., three barriers), as it was the only innovation that required health behavior change in patients and caregivers.

### Worked example of a funded project (Project 01)

#### Overview of innovation – Artificial intelligence for glaucoma diagnosis

Glaucoma is an eye disease associated with raised intraocular pressure (8). If untreated, glaucoma can result in irreversible blindness (8). Diagnosis of glaucoma is typically complicated and requires a battery of tests conducted over several visits (9). However, recent progress in artificial intelligence in health care has promised to change the diagnostic landscape of glaucoma (9). Innovation proposal 01 proposed the use of an artificial intelligence platform in local glaucoma clinics. The proposed platform can diagnose and determine the severity of glaucoma from a single optical coherence tomography scan of the optic nerve head (10). The project team has previously established its predictive accuracy and proposed to conduct a pilot study to provide real-world performance indicators (11).

#### Potential value to the institution

The proposal scored well for the following “value to the institution” attributes: “population impact,” “evidence-based,” “health outcomes,” “prevention,” “access,” “affordability,” “capability building,” and “sustainability.” Glaucoma is one of the most common ophthalmologic conditions in Singapore. The prevalence of glaucoma among Singapore adults was estimated to be 3.6 percent and is projected to increase as the population ages (12). Hence, the IAU estimated that the proposed technology will benefit more than 100,000 patients in a 5-year period and scored it well for “population impact.” The proposed innovation also scored well for the “evidence-based” attribute. A systematic review revealed that deep learning in optical coherence tomography for glaucoma diagnosis is efficient and accurate (13). The project team has also provided preliminary findings that showed high diagnostic accuracy of the proposed platform was 92.0 ± 2.3 percent (11). Our purposive search also revealed that innovation will likely confer health benefits to patients and score it well for “health outcomes.” Improved diagnostic accuracy meant more glaucoma patients can be adequately treated to prevent visual field loss (8). Finally, the proposal scored well for “economic productivity” as it is likely that the innovation will bring about economic benefits to both patients and the hospital. Time spent by patients and caregivers is likely to be reduced, improving economic productivity in patients and caregivers (14). The platform will also allow junior doctors to perform glaucoma diagnosis and free up the senior doctors for more complex tasks.

#### Novelty

The IAU found similar innovations in literature. Maetschke et al. (15) proposed a deep-learning technique using optical coherence tomography three-dimensional volumetric scans to classify eyes as healthy or glaucomatous, with an accuracy of 94 percent. Ran et al. (16) developed a deep-learning model using 6,921 optical coherence tomography scans with an accuracy of 91 percent upon primary validation. However, the study team was able to provide sound justifications on why their proposed platform should be funded despite the availability of similar substitutes. First, their innovation has a diagnostic accuracy that is comparable to those reported in literature, and has the additional advantage of been validated using local data where it will be applied in practice (11). Next, their innovation could be provided to the local hospitals free of charge as it was a hospital-funded initiative.

#### Potential barriers to implementation

One potential barrier for implementation highlighted by the IAU was the “black-box” effect of deep-learning algorithms (9). Decision-making processes of deep-learning systems are not intuitively interpretable to clinicians; hence, clinicians may be wary or sceptical of the technology. In the written feedback, IAU suggested that appropriate effort should be invested by the study team into the education of patients and clinicians to increase their understanding of the deep-learning system. Further qualitative research to elucidate the best scheme for integrating the proposed platform into current clinical practice in a way that is acceptable to clinicians may also be beneficial.

## Discussion

We developed a health innovation assessment tool that is aligned to our institutional priorities. The assessment tool can be applied to different types of health innovations and allow them to be compared using a common standard. We believe that our tool is useful in guiding funding decisions in panellists who may also consider other aspects of the innovation not within our assessment criteria.

The background reasoning that underpins the design of our assessment tool is similar to that used in traditional health technology assessment (HTA) (17). However, traditional HTA is time-consuming and is not suitable for prospectively evaluating a large number of health innovations under tight time constraints (17). A number of decision support tools for the rapid assessment of health innovations in hospitals has been described in literature. One of the most well-known tools is the “mini-HTA,” which is an open-ended questionnaire containing questions about the technology, patient, organization, and financial aspects (18). Sampietro-Colom et al. (19) further adapted the mini-HTA by classifying twelve mini-HTA variables into a two-dimensional value/risk visual scoring system. Blythe et al. (4) created a multicriteria decision analysis tool to evaluate six fields of healthcare provision – return on investment, capacity, outcomes, safety, training, and risk. In our study, we identified attributes of values based on our institutional priorities. The attributes identified in our assessment tool aligned with major criteria listed in previously published frameworks (20). In a review by Cruz-Riveria et al. (20), the most common criteria listed for health systems impact were quality of care, service delivery, cost containment, cost effectiveness, health information management, and evidence-based practice.

As the institution may assign dissimilar importance to the attributes of values, we allocated weighted scores to the value attributes based on a preference survey of the institution’s innovation leaders. We recognized that the attributes values by the institution and their preference weights is dependent on the application and may change with time and context; hence, it will be essential to review and update these attributes regularly. We did not allocate scores to the “novelty” or ‘potential barriers to implementation’ components. This is because these components were not designed to rank innovations, but to foster further discussions on the possibility to modify existing technologies to address the healthcare gap or pragmatic considerations of overcoming potential barriers. Hence, we acknowledge that our assessment tool has an important shortcoming. The apparent numerical precision of the “value to the institution” scores might result in panellists awarding proposals based on these scores alone without giving due consideration to the other two components. To minimize this, we presented the scores of the value component using a visual analog scale as opposed to providing the actual numerical scores of each project (see Supplementary Figure 1).

Our assessment tool considered evidence from multiple sources, including published literature, preliminary data, and stakeholders’ input. We used a purposive approach to conduct our literature search as opposed to a systematic review with pre-defined keywords (7). This is because purposive searches offered greater flexibility to review a varied array of relevant but dissimilar papers, allowing us to reflect broadly on each attribute before drawing conclusions (7). We classified the potential barriers to implementation based on the Theoretical Domains Framework (6), which identified barriers related to individual level change and provided a good coverage of potential implementation problems. Another framework that is commonly employed for evaluation of research and innovation implementation is the Consolidated Framework for Implementation Research (21), which provides a broad overview of specific constructs related to healthcare interventions. Moving forward, we hope to incorporate components from other implementation frameworks into our assessment tool for a more comprehensive evaluation of potential barriers (22).

Our work has limitations. First, we designed the value component based on prevailing stated institutional priorities. This meant that potential advantages not aligned with the stated institutional priorities are excluded; for example, a unique feature of one proposed innovation was its benefits to the environment as it was constructed using fully biodegradable materials. In addition, our assessment tool focused on indicators associated with the health systems impact and patient outcomes. Research-related indicators such as number of publications were deemed to be less crucial for the successful implementation of a health innovation (20). Second, purposive literature searches, while offering flexibility to assess a diverse array of relevant papers, offer less assurance of a balanced perspective compared to systematic searches (7). To minimize evidence selection bias, two researchers were tasked to review the literature independently for each innovation proposal. Last, given the lack of actual data and meagre 3-week timeframe for impact assessment, we intentionally omitted any costing or cost-effectiveness modeling in our proposed tool.

## Conclusions

The decision process for funding healthcare innovations is complex and assessment tools can be helpful in directing the allocation of scarce resources to competing innovations (20). The main advantage of our tool is that it facilitates systematic and transparent assessment; nonetheless, the tool is not designed to provide precise measurements of impact and some subjectivity will remain. While our tool is currently aligned with our institutional priorities, it can be easily adapted to assess healthcare innovations in other settings.

## Supplementary material

For supplementary material accompanying this paper visit https://doi.org/10.1017/S0266462323000028. click here to view supplementary material
