# Supplementary material for: A decision-support tool for funding health innovations at a tertiary academic medical center
Source: Int J Technol Assess Health Care. 2023 Feb 13;39(1):e11. doi: 10.1017/S0266462323000028 (PMC11574547; doi:10.1017/S0266462323000028)
Supplement: Supplementary file 1 [file S0266462323000028sup001.docx]

**Supplementary Appendix**

**Supplementary Table 1. Relative importance of value to institution attributes to key opinion leaders (n = 12)**

| **Attribute** | **Rank** | **Mean score out of five (sd)** |
| --- | --- | --- |
| **Health outcomes** | 1 | 4.25 (0.75) |
| **Patient-centred** | 1 | 4.25 (0.75) |
| **Access** | 2 | 4.00 (0.60) |
| **Sustainability** | 2 | 4.00 (0.60) |
| **Safety** | 3 | 3.90 (0.80) |
| **Evidence-based** | 4 | 3.85 (1.05) |
| **Affordability** | 5 | 3.75 (0.95) |
| **Capability building** | 5 | 3.75 (0.95) |
| **Economic productivity** | 5 | 3.75 (0.95) |
| **Population impact** | 5 | 3.75 (1.05) |
| **Prevention** | 6 | 3.65 (0.90) |
| **Professional development** | 7 | 2.65 (1.15) |
|  |  |  |

Abbreviations used: sd, standard deviation

**Supplementary Table 2. Description of purposive searches conducted by the IAU**

| **Attribute** | **Short description** | **Databases/Search Engine** | **Scope of purposive search** | **Other forms of data input** |
| --- | --- | --- | --- | --- |
| 1. **Potential value to the institution** | | | | |
| **Access** | The innovation can improve access to healthcare services for patients | - PubMed - Cochrane Library - Google Scholar | - Published literature of similar innovations, with access to healthcare services as a study outcome. - Level of evidence was rated using the GRADE quality scale. | - Preliminary data provided by the innovation teams |
| **Affordability** | The innovation can eventually result in health savings for the institution and/or the healthcare system. | None | None | - Projected cost calculations provided by the innovation teams, which are assessed by the IAU |
| **Capability building** | The innovation can foster further research and innovation partnerships and/or has the potential to be scaled beyond the SingHealth Duke-NUS AMC. | - PubMed - Cochrane Library - Google Scholar - Google search engine | - Published literature on clinical gaps in other local healthcare institutions locally or globally, that might be addressed by the proposed innovation - Examples of commercialisation /patent of similar innovations | - Evidence of collaborative partnerships fostered provided by the innovation team |
| **Economic productivity** | The innovation can improve the productivity of patient or caregivers (e.g., shorter downtime). | - PubMed - Cochrane Library - Google Scholar | - Published literature on similar innovations, with economic productivity as a study outcome. - Level of evidence was rated using the GRADE quality scale. | - Preliminary data provided by the innovation teams |
| **Evidence-based** | There is existing evidence that the innovation can address the stated healthcare problem | - PubMed - Cochrane Library - Google Scholar | - Published literature on similar innovations and impact on stated problem/healthcare gap as a study outcome. - Level of evidence was rated using the GRADE quality scale. | - Preliminary data provided by the innovation teams |
| **Health outcomes** | The innovation can reduce morbidity and/or mortality | - PubMed - Cochrane Library - Google Scholar | - Published literature on similar innovations, with morbidity/mortality as a study outcome. - Level of evidence was rated using the GRADE quality scale. | - Preliminary data provided by the innovation teams |
| **Patient-centred** | The innovation can address the specific needs of patients and/or caregivers | - PubMed - Cochrane Library - Google Scholar - Google search engine | - Published literature (surveys, structures interviews, focus groups) suggesting gap in patient/caregiver needs - Published reviews and expert opinion papers - Local publications (e.g., news reports, institutional reports) | - Preliminary data provided by the innovation teams |
| **Population impact** | The innovation can impact many patients | - PubMed - Cochrane Library - Google Scholar - Google search engine | - Published literature on local or global epidemiology of stated clinical problem - Local government or census websites | - Preliminary data provided by the innovation teams - Number of patients with the stated clinical problem seen annually, obtained from the SingHealth electronic clinical database |
| **Prevention** | The innovation can directly prevent health deterioration or disease occurrence in the future | - PubMed - Cochrane Library - Google Scholar | - Published literature on similar innovations, with disease prevention as a study outcome. - Level of evidence was rated using the GRADE quality scale. | - Preliminary data provided by the innovation teams |
| **Professional development** | The innovation can enable staff to gain new skills. | None | None | - Evidence of staff development opportunities provided by project teams, which are assessed by the IAU |
| **Safety** | The innovation can improve safety for patients and/or staff | - PubMed - Cochrane Library - Google Scholar | - Published literature on similar innovations, with patient/healthcare worker safety as an outcome measure. - Level of evidence was rated using the GRADE quality scale. | - Preliminary data provided by the innovation teams |
| **Sustainability** | The innovation is likely to be adopted in a continued manner in the future in the local healthcare setting | None | None | - Evidence of buy-in from relevant stakeholders provided by project team in submitted proposals, which are assessed by the IAU - Endorsement by clinical chairperson and support from centre representatives |
| 1. **Novelty** | | | | |
| **Novelty** | The innovation offers a novel solution to the existing healthcare problem | - PubMed - Cochrane Library - Google Scholar - Google search engine | - Published literature with similar innovations - Published literature describing alternative solutions to the existing healthcare problem - Commercial websites offering similar innovations - Local and international non-peer reviewed publications (e.g., news reports, institutional reports, presentations) describing similar innovations - Local and international non-peer reviewed publications (e.g., news reports, institutional reports, presentations) describing alternative solutions to the existing healthcare problem | None |
| 1. **Potential barriers to implementation** | | | | |
| **Social** | There are foreseeable social barriers that may prevent implementation of the innovation | - PubMed - Cochrane Library - Google Scholar - Google search engine | - Published literature describing the implementation of similar innovations to identify social issues that were encountered - Reviews and expert opinions of similar innovations - Local publications (e.g., news reports, institutional reports) relevant to the innovation | - Preliminary evidence provided by project teams |
| **Environmental** | There are potential limitations in the present resources that may prevent the implementation of the innovation | - PubMed - Cochrane Library - Google Scholar - Google search engine | - Published literature describing the implementation of similar innovations to identify environmental resources required - Reviews and expert opinions of similar innovations - Local publications (e.g., news reports, institutional reports) relevant to the innovation | - Preliminary evidence provided by project teams - Discussion with key opinion leaders in the SingHealth Duke-NUS AMC |
| **Intention** | Relevant stakeholders demonstrate a certainty of commitment to implement of the innovation | None | None | - Evidence of buy-in from relevant stakeholders provided by project team in submitted proposals, which are assessed by the IAU |
| **Professional roles** | The stakeholders involved are the appropriate group to implement the innovation | - PubMed - Cochrane Library - Google Scholar | - Published literature describing the implementation of similar innovations to identify stakeholders involved - Reviews and expert opinions of similar innovations | - Evidence of buy-in from relevant stakeholders provided by project team in submitted proposals, which are assessed by the IAU |
| **Belief about consequences** | Expectations of the impact of the proposed innovation is reasonable | - PubMed - Cochrane Library - Google Scholar | - Published literature on similar innovations and impact on stated problem/healthcare gap - Reviews and expert opinions of similar innovations | - Timeline and milestones provided by the project team |
| **Belief about capabilities** | The stakeholders are empowered to implement the innovation. | None | None | - Discussion with key opinion leaders in the SingHealth Duke-NUS AMC |
| **Goals** | Proper priority setting and action planning for implementation are in place. | None | None | - Timeline and milestones provided by the project team |
| **Emotions** | The stakeholders have no emotional barriers preventing the implementation of the innovation. | - PubMed - Cochrane Library - Google Scholar | - Published literature describing the implementation of similar innovations to identify stakeholders involved and any potential emotional barriers | - Evidence of buy-in from relevant stakeholders provided by project team in submitted proposals, which are assessed by the IAU |
| **Knowledge** | Significant advancement of knowledge for the users is required before the innovation can be implemented | - PubMed - Cochrane Library - Google Scholar | - Published literature describing the implementation of similar innovations to identify any potential knowledge barriers | - Methodology provided by project teams |
| **Skills** | Specialized training for users is required before the innovation can be implemented | - PubMed - Cochrane Library - Google Scholar | - Published literature describing the implementation of similar innovations to identify any potential skill barriers | - Methodology provided by project teams |

Abbreviations used: AMC, academic medicine centre; GRADE, Grading of Recommendations, Assessment, Development and Evaluations; IAU, impact assessment unit.

**Supplementary Figure 1. Example of (a) feedback provided to innovation teams, and (b) impact assessment report for independent panel of assessors.**

**
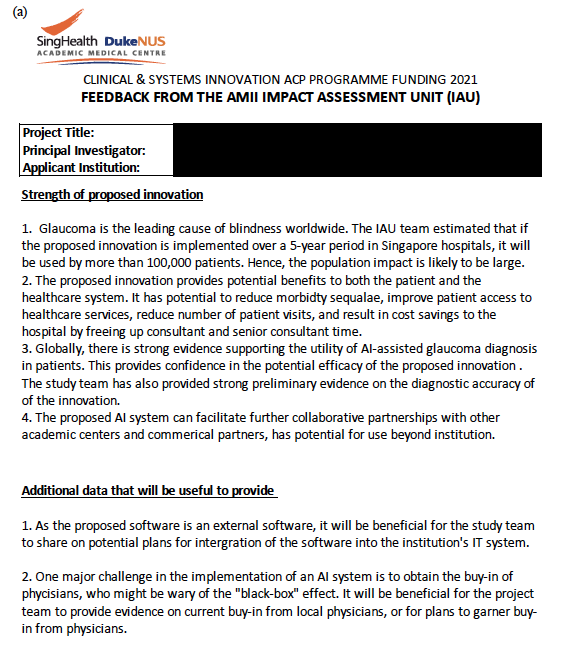
**

**
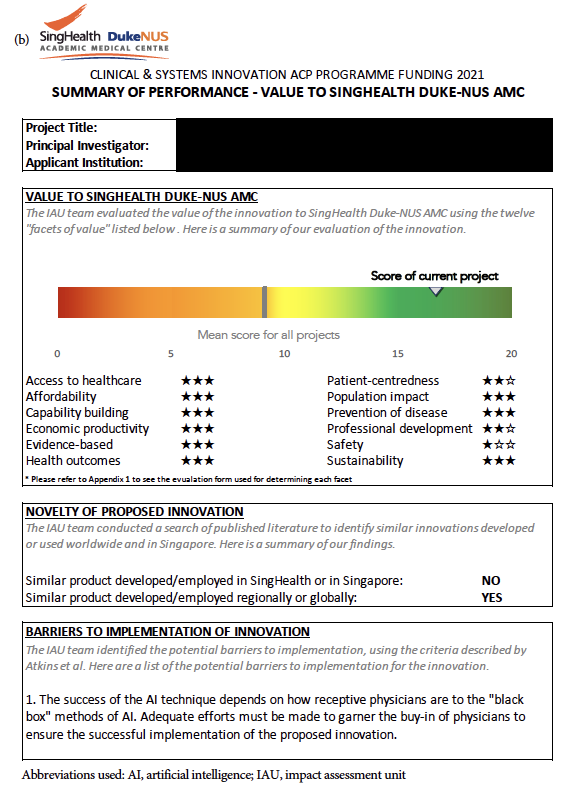
**
